# Polysubstance use in the U.S. opioid crisis

**DOI:** 10.1038/s41380-020-00949-3

**Published:** 2020-11-13

**Authors:** Wilson M. Compton, Rita J. Valentino, Robert L. DuPont

**Affiliations:** 1 U.S. Department of Health and Human Service, National Institute on Drug Abuse, National Institutes of Health, Bethesda, MD USA; 2 Institute for Behavior and Health, Inc., Rockville, MD USA

**Keywords:** Addiction, Neuroscience

## Abstract

Interventions to address the U.S. opioid crisis primarily target opioid use, misuse, and addiction, but because the opioid crisis includes multiple substances, the opioid specificity of interventions may limit their ability to address the broader problem of polysubstance use. Overlap of opioids with other substances ranges from shifts among the substances used across the lifespan to simultaneous co-use of substances that span similar and disparate pharmacological categories. Evidence suggests that nonmedical opioid users quite commonly use other drugs, and this polysubstance use contributes to increasing morbidity and mortality. Reasons for adding other substances to opioids include enhancement of the high (additive or synergistic reward), compensation for undesired effects of one drug by taking another, compensation for negative internal states, or a common predisposition that is related to all substance consumption. But consumption of multiple substances may itself have unique effects. To achieve the maximum benefit, addressing the overlap of opioids with multiple other substances is needed across the spectrum of prevention and treatment interventions, overdose reversal, public health surveillance, and research. By addressing the multiple patterns of consumption and the reasons that people mix opioids with other substances, interventions and research may be enhanced.

## Introduction

The U.S. opioid crisis exerts a major impact on health and social outcomes. Since 2005, deaths due to opioid overdoses exceed 500,000 [1], and declines in overall U.S. life expectancy are at least partly explained by overdose mortality [2, 3]. Morbidity caused by opioids includes increasing infectious disease (particularly HIV, hepatitis, and endocarditis), sleep disorders, affective disorders, and increasing neonatal opioid withdrawal, among other outcomes [4–9]. Overall economic impact from opioid misuse was estimated at $500 billion per year in 2017 [10].

In response, prevention and treatment practices and policies have been implemented widely [5, 11]. These include expanding access to medications for opioid use disorder (OUD) [12–15], reducing opioid prescribing [16–19], increasing access to naloxone [20–24], improving public health surveillance [16], increasing access to harm reduction programs such as syringe services [16, 25, 26], and major investments in research [27, 28]. All of these interventions target opioid use, misuse, and addiction. However, if the “opioid crisis” includes multiple substances, the opioid specificity of current prevention and treatment interventions may limit their ability to address the broader problem of polysubstance use involving opioids. To have the maximum impact on this multifaceted crisis, it is important to understand the overlap of opioids with other substances.

Overlap of opioids with other substances can include shifts across the lifespan and simultaneous co-use of substances. Use of substances across a broad time frame (i.e., past year or lifetime) is typical for epidemiological studies, which less commonly assess specific co-use patterns within specific drug using occasions. However, the primary focus of this Perspective is on the issue of simultaneous co-use of an opioid with another substance, which could include nicotine, alcohol, other opioids including prescribed opioids, benzodiazepines (and other sedatives), stimulants including cocaine and amphetamine-related compounds, and other categories.

There are multiple reasons people use other substances with opioids: enhancement of the high (additive or synergistic reward), compensation for undesired effects of one drug by taking another, compensation for negative internal states, or a general predisposition for all substances. The possibility that consuming multiple substances in combination has additive or synergistic pharmacological effects particularly demands study. Fortunately, we are now at a point where advances in research methods allow us to study these kinds of complexities, moving addiction science beyond the typically single-drug models of the past.

In this Perspective, we summarize overlaps of opioid use and addiction with use of and addiction to other substances from viewpoints of population science and then neuroscience. In each section we address questions of why nonmedical opioid users also commonly use other drugs, and what are health consequences of simultaneous use of multiple drugs, including their role in overdose deaths.

## Population studies

Epidemiological studies dating back at least several decades confirm that persons with OUD are likely to have used multiple substances, not just opioids. The Epidemiologic Catchment Area data from the early 1980s showed that, on average, heroin users consumed 5.0 other illicit substances (out of seven categories assessed and excluding tobacco or alcohol) and users of opioids other than heroin consumed 5.8 others [29]. Results from observational studies of returning Vietnam Veterans and samples of heroin users in Miami and California confirm other drug use as a prominent risk factor for subsequent heroin use and overlapping co-use of heroin with other substances [30–34].

More recent studies also have documented that opioid use overlaps with other substances and that OUD often co-occurs with other substance use disorders [35–38]. A 2012–2013 U.S. general population study found that more than 90% of individuals with OUD used more than two other substances within the same year, and over 25% had at least two other substance use disorders along with OUD [39]. Those with more substance use disorders were more likely to be younger, male, and single and were more likely to have initiated opioid use at an earlier age [39]. Finally, a notable indicator of simultaneous multi-substance use is that the majority of opioid-related overdose deaths involve multiple substances (see “Health and Safety Consequences” below).

While overlap with other substances and substance use disorders is common among those with OUD, the patterns may vary [40]. Recent evidence shows an increasing rate of methamphetamine use among adults in the U.S. admitted to treatment programs for heroin addiction [41]. The implication is that overlap of opioids with other substances is common but can vary across time, both within an individual and within populations of users.

**Fig. 1:**
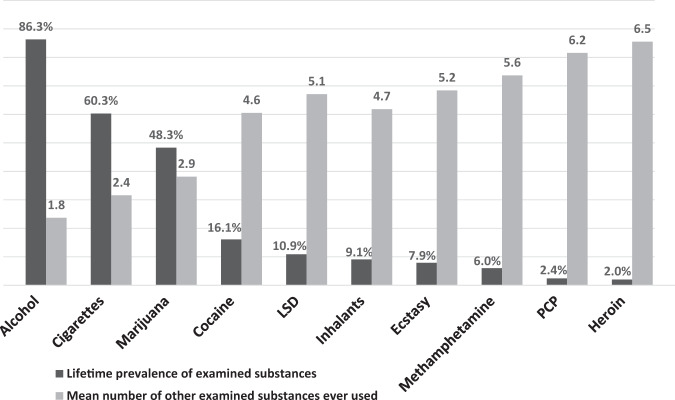
Overlap of substances used across the lifetime. Weighted lifetime prevalence of substance use and mean number of other substances ever used by adults age 18 and older in the United States (*n* = 51,000; Source: 2018 U.S. National Survey on Drug Use and Health [adapted from Eric Wish, University of Maryland, Center for Substance Abuse Research]).

While the focus of this Perspective is on polysubstance use in the context of opioids, it is important to consider that polysubstance use frequently occurs with all recreational drugs, whether their use is legal or illegal. Cigarette smokers are far more likely to drink alcohol than non-smokers. Marijuana users are far more likely to use opioids nonmedically than people who do not use marijuana. This pattern is seen in all ages and drug-using populations [42]. Further, as a general principal, the more widely a drug is used, the higher the percentage of users who do not use other drugs; and, the less widely used, the more likely a drug is to be used with other drugs (Fig. 1). While the reasons for this increasing overlap in substances that are less frequently consumed is not totally clear, some of it may relate to the drug use trajectories where substances are added to one another in a progression. Because less frequently consumed substances are rarely the first used, it is typical that less frequently consumed substances would have a higher degree of overlap over the life course than the more commonly consumed. This principal may help explain the high frequency of overlap of heroin use with other drug use and the lesser, but still significant, overlap of prescription opioid misuse (which is much more common than heroin use) with other drug use.

### Compensation for negative internal states

Polysubstance use, including when it involves opioids, may be a way to compensate for negative internal states associated with psychiatric disorders (maladaptive self-medication). Mental illnesses, especially mood disorders, often co-occur with opioid use and use disorders [35, 37]. Of the 9.6 million American adults who misused opioids in 2018, 45.8% (4.4 million) had a mental illness in the past year, and 17.3% (1.7 million) had a serious mental illness in the past year [43]. The rates of co-occurring mental illness are even higher for those with a past year OUD, with 66.7% having any mental illness in the past year and 28.6% having serious mental illness in the past year [43]. In 2018, 17.3% of the adults who had misused opioids (1.6 million people) reported having serious thoughts of suicide, 6.6% (0.6 million) made suicide plans, and 3.2% (0.3 million) attempted suicide [43]. In one study individuals with substance use disorders involving multiple drugs were more likely to have other mental illnesses than were those with just OUD [39]. In particular, polysubstance use may have contributed to post-traumatic stress disorder because of its deleterious effects on neuroendocrine stress-response systems [39].

OUD is also highly comorbid with pain. Chronic pain and the associated emotional distress may dysregulate the brain’s reward and stress circuitry, raising the risk for opioid misuse and OUD [44]. One study estimates that 10% of patients treated for chronic pain misuse prescription opioids [44]. The use of other drugs including alcohol, cannabis, or sedatives is common in individuals with chronic pain. Although the prevalence of chronic pain is positively correlated with the number of comorbid substance use disorders, it was not found to be a significant factor in the prevalence of comorbid substance use disorders for individuals with OUD [45]. One possible explanation for this was that a greater proportion of the chronic pain/OUD group were females who are less likely to be polysubstance users. Consistent with this, in a study of individuals under treatment for substance use disorder that have chronic pain, polysubstance use was associated with pain interference only in males with alcohol use disorder [46]. Chronic pain was also found to be associated with mental health disorders, particularly mood disorders, in individuals with substance use disorders and this was highest for individuals with both OUD and chronic pain, implying important interactions between mental illness, pain, and OUD [46].

### Compensation for other drug effects

An incentive for polysubstance use is to compensate for impairments associated with opioid use. Just as users may perceive that co-use of stimulants ameliorates alcohol impairment, people who use opioids may take stimulants to compensate for opioids’ sedating effects. One of the motivations for use of prescription opioids, stimulants, and benzodiazepines has been to “increase or decrease the effects of other drugs” [37, 47, 48]. For example, the addition of cocaine to decrease the dose of heroin and degree of physical dependence has been reported by out-of-treatment users and by methadone patients [49]. Such combinations of heroin and cocaine may be sought because of both the rewarding effects and as compensation for adverse opioid effects [50].

### Health and safety consequences

Toxicity can be increased through pharmacokinetic or pharmacodynamic interactions as described in the “Neuroscience” section below, and drug combinations involving opioids may be particularly dangerous from the standpoint of overdose risk [51, 52]. Detailed analyses of polysubstance use in Florida identified multiple causative substances in 93% of the 1743 fentanyl-related overdose deaths in 2017 [53]. Use of other substances appears to play a role in overdose from heroin [54–56], and combinations of opioids with other substances, especially sedatives and respiratory depressants such as alcohol and benzodiazepines, increase the risk of overdose [51, 57–59].

Overdose research typically reports each category of substances separately, without accounting for overlap. For instance, the primary report on drug overdose deaths from the National Center for Health Statistics reported on all overdose deaths combined and deaths from: (1) any opioid or (2) heroin, (3) natural and semisynthetic opioids, (4) methadone, and (5) synthetic opioids other than methadone [1]. Yet, deaths involving more than one category are coded in all that are included, perhaps masking important trends in which multiple drugs may be the culprit more than one specific drug.

Because of its very high potency and its ubiquity in mixtures with heroin and sometimes with other drugs such as cocaine or counterfeit prescription medications, polysubstance use with fentanyl may be responsible for the high fatality rate in many areas of the United States. In 2016, 79.7% of overdose deaths in the U.S.A. involving synthetic opioids other than methadone (i.e., fentanyl-related compounds) also involved other substances, particularly other opioids, cocaine, benzodiazepines, alcohol, psychostimulants, and antidepressants [60]. Purveyors of the drugs (i.e., the vector) may be responsible for some of the polydrug use found in overdose decedents [5]. It may or may not have been the explicit choice of a user to combine substances, and reasons for the multiple drug use may include both intentional co-ingestion as well as adulteration of the illicit drug supply [61].

Benzodiazepines are an important category to consider. By themselves, benzodiazepines have a generally limited ability to result in overdose death [62]; however, the combination with opioids is increasingly detected in overdose deaths, suggesting synergistic effects [51]. Reasons for this increased lethality of the combinations are not fully understood and may vary depending on the specific opioid and benzodiazepine involved [63].

Polysubstance use may also contribute to risk of traumatic injury, infectious disease risk, and other health consequences [64]. Between 40 and 60% of patients seen in trauma centers have alcohol or other drugs in their system, and, in one study, presence of cocaine, marijuana, or opioids in the blood was significantly more likely among injured patients with acute alcohol intoxication than in those without alcohol intoxication [65]. Furthermore, settings where patients with injuries due to polysubstance consumption are treated (including emergency and inpatient settings) can be useful in providing opportunities for intervention and engagement in care that is needed over the long term.

## Neuroscience studies

### Convergent reward mechanisms

A neurobiological basis for polysubstance use is that combining drugs that converge on a common circuit at different points can produce additive or synergistic rewarding effects. Synergistic pharmacological interactions occur with drugs that have different mechanisms but a common end consequence, such as increasing dopamine (DA) release in the nucleus accumbens (NAC). With synergistic interactions, the effect of the combination is greater than the sum of the separate drug effects. This contrasts with additive pharmacological interactions, which occur between drugs that share a common mechanism and would result in an effect that is equal to the sum of both agents.

**Fig. 2:**
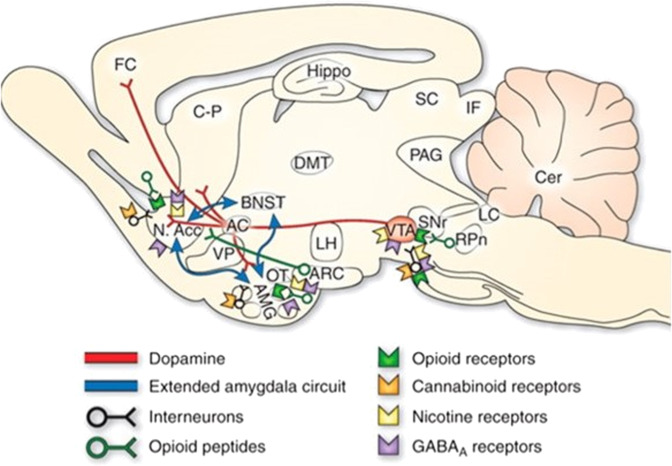
Rat brain neurochemical neurocircuits in drug reward (From [101]). Sagittal section through a representative rodent brain illustrating the pathways and receptor systems implicated in the acute reinforcing actions of drugs of abuse. Cocaine and amphetamines activate the release of dopamine in the nucleus accumbens and amygdala through direct actions on dopamine terminals. Opioids activate opioid receptors in the VTA, nucleus accumbens, and amygdala through direct or indirect actions via interneurons. Opioids facilitate the release of dopamine in the nucleus accumbens by an action either in the VTA or the nucleus accumbens, but also are hypothesized to activate elements independent of the dopamine system. Alcohol activates g-aminobutyric acid-A (GABAA) receptors or GABA release in the VTA, nucleus accumbens, and amygdala by either direct actions at the GABAA receptor or through indirect release of GABA. Alcohol is hypothesized to facilitate the release of opioid peptides in the VTA, nucleus accumbens, and central nucleus of the amygdala. Alcohol facilitates the release of dopamine in the nucleus accumbens through an action either in the VTA or the nucleus accumbens. Nicotine activates nicotinic acetylcholine receptors in the VTA, nucleus accumbens, and amygdala, either directly or indirectly, through actions on interneurons. Cannabinoids activate cannabinoid CB1 receptors in the VTA, nucleus accumbens, and amygdala. Cannabinoids facilitate the release of dopamine in the nucleus accumbens through an unknown mechanism either in the VTA or the nucleus accumbens. The blue arrows represent the interactions within the extended amygdala system hypothesized to have a key function in drug reinforcement. The medial forebrain bundle represents ascending and descending projections between the ventral forebrain (nucleus accumbens, olfactory tubercle, septal area) and the ventral midbrain (VTA) (not shown in figure for clarity). AC anterior commissure, AMG amygdala, ARC arcuate nucleus, BNST bed nucleus of the stria terminalis, Cer cerebellum, C-P caudate-putamen, DMT dorsomedial thalamus, FC frontal cortex, Hippo hippocampus, IF inferior colliculus, LC locus coeruleus, LH lateral hypothalamus, N Acc. nucleus accumbens, OT olfactory tract, PAG periaqueductal gray, RPn reticular pontine nucleus, SC superior colliculus, SNr substantia nigra pars reticulata, VP ventral pallidum, VTA ventral tegmental area.

The rewarding effects of substances are mediated by ventral tegmental area (VTA)-dopaminergic projections to the NAC [66]. The action of this pathway can be enhanced by several mechanisms, including by directly or indirectly increasing activity of VTA-DA neurons, increasing terminal DA release, blocking DA reuptake or enhancing the postsynaptic actions of DA on NAC neurons. Preclinical studies in rodents have identified the neuronal and neurochemical actions of drugs on this circuit (see Fig. 2).

Opioids are distinct from other rewarding substances through their actions at specific opioid receptors. Studies in animals indicate that activation of μ-opioid receptors on GABA-VTA cells disinhibits DA neurons and increases their activity and DA function in the NAC [67]. When opioid receptors are maximally occupied, the addition of another opioid has no further effect. However, combinations with stimulants that increase synaptic levels of DA or that enhance DA terminal release results in a synergistic effect on DA release that is greater than the effect of either alone [68, 69].

Preclinical studies demonstrated that cocaine enhances DA levels primarily through inhibition of the DA transporter, whereas amphetamine-like stimulants both inhibit the transporter and also directly increase vesicular release [70]. The net effect of both is to increase DA in the NAC. For example, cocaine increases inhibitory GABA transmission from the prefrontal cortex to VTA-GABA neurons, an effect that would disinhibit VTA-DA neurons through a mechanism that is distinct from that mediated by opioid receptors [71]. In addition to its primary effect to increase terminal DA release, amphetamine inhibits monoamine oxidase and enhances tyrosine hydroxylase, which would have the net effect of increasing DA extracellular levels, also in ways that are distinct from opioid mechanism [72].

Although there is substantial evidence for synergistic effects of stimulant/opioid combinations on DA release in animals, self-administration of the combination reflected an additive interaction, i.e., it was not greater than the sum of the individual drug effects in animals [73]. In human laboratory studies the effects are mostly additive but this is dose-related [74]. For example, certain dose combinations increased ratings of “high” and “liking” and cocaine administered to methadone-maintained individuals produced greater subjective ratings of “high” and “liking” [74, 75]. In drug discrimination studies in non-human primates the drug combination produces a unique discriminative stimulus that shares characteristics with the individual drugs but does not completely overlap them [76].

Notably, the actions of stimulants are not limited to DA systems. They also increase the activation of the other monoamine systems, serotonin and norepinephrine [70]. The bias toward a particular monoamine system depends on the specific stimulant. For example, MDMA (ecstasy) has a greater effect on serotonin than amphetamine, which is biased toward DA and norepinephrine systems [77]. The enhancement of noradrenergic function by stimulants may further drive opioid use in an effort to compensate for or balance the arousal response.

Benzodiazepines are often co-administered with opioids. Because they are allosteric modulators of GABA A receptors (GABAAR), benzodiazepines might be predicted to oppose the disinhibitory effects of opioids in the VTA. However, although both GABA and DA neurons in the VTA express GABAAR, benzodiazepines bind to those containing the α1 subunit, which are localized to VTA-GABA neurons and are lacking in VTA-DA neurons [78–80]. The resulting inhibition of VTA-GABA neurons would be additive to the acute action of opioids. In human studies, benzodiazepines enhance the subjective effects of opioids, including “high” and “liking” [63]. Benzodiazepines are commonly prescribed along with opioid analgesics and are commonly misused by those who use illicit opioids [63, 81], and the combination has been implicated in increasing the risk for overdose [51, 57, 63, 81]. Both benzodiazepines and opioids inhibit respiration so that the effects of the combination may be additive. Although mechanisms underlying this interaction have not been elucidated, in animal models pretreatment of a benzodiazepine potentiated opioid-induced brain hypothermia, but not hypoxia, whereas co-administration of the two potentiated the opioid-induced decrease in oxygen levels in brain [82].

The complex rewarding effects of nicotine are mediated by multiple receptors in the VTA and NAC, although its actions at the β2-nAChR subtype are considered to be integral to the rewarding effects [83]. Unlike opioids, nicotine directly depolarizes VTA-DA neurons [84]. This would produce a greater magnitude of neuronal activation than disinhibition alone and would synergize with opioid disinhibition of the same neurons. In addition to increasing DA discharge rate, nicotine changes the pattern of discharge to favor a phasic or bursting mode [85]. This is mediated by α7-nAChR enhancement of glutamatergic signaling from the prefrontal cortex to VTA-DA neurons [86, 87]. DA bursting promotes the formation of associations between stimuli and rewards and this may be the basis for reward-enhancing effects of nicotine in combination with other substances [88, 89].

### Gateway priming

Typically, the term “gateway drug theory” refers to use of tobacco and alcohol preceding marijuana, followed by “harder drugs” like cocaine or opioids, and most who misuse opioids have already used legal drugs and marijuana [29]. An important consideration of the theory is that the gateway drug is often used during adolescence. This is a window of brain development during which perturbations such as drug exposure can produce unique and enduring effects.

While multiple studies document overlaps in substance use onset [42, 90], mechanisms for a gateway potential for nicotine have perhaps best been delineated. Neurobiological evidence suggests that, independent of its own rewarding effects, nicotine can intensify rewarding effects of other substances and non-drug rewards [89, 91]. In addition to the ability of nicotine to favor phasic DA neuronal discharge, this may also be attributed in part to its ability to increase tyrosine hydroxylase activity [92, 93]. Nicotine’s actions as a histone deacetylase inhibitor could prime a cocaine response by enhancing cocaine-related histone-4 acetylation of the FosB gene, and the hyperacetylation of other histones by nicotine would further enhance gene expression [91]. Analyzing data from two cohort studies, Kandel and Kandel found that cocaine dependence was highest in users who had first smoked cigarettes and that concurrent smoking around the time of cocaine initiation was associated with more persistent cocaine use and addiction—consistent with the priming effect they found in their animal model. Conversely, cocaine does not appear to prime the nicotine response [91]. Although this novel hypothesis is compelling, it awaits confirmation by other studies and the linkages to later addiction to opioids are not fully established. Future studies identifying these effects are necessary for understanding the potential for licit drug administration during adolescence to accelerate progression to illicit drug use.

### Neuroplasticity

In addition to acute actions described above, all drugs of abuse produce long-term plasticity that contributes to compulsive behaviors that characterize substance use disorder. They do so through multiple mechanisms, and a description of this is beyond the scope of this Perspective. However, a shared mechanism through which most drugs of abuse induce enduring neuroadaptations is by increasing the AMPAR/NMDAR ratio, resulting in enhanced excitatory transmission onto VTA-DA neurons [70, 94]. The mechanisms by which individual drug classes produce their effects and whether these are specific to individual classes is a topic of future investigation.

## Implications for research

The study of multiple substances requires assessment of both individual and combined effects. Therefore, when multiple substances are involved a general issue is the difficulty of framing meaningful research hypotheses and selecting from a broad range of possible analyses. The National Institutes of Health provides guidance through a specific funding opportunity announcement on the importance of both observational and treatment development research on polysubstance use (see: https://grants.nih.gov/grants/guide/pa-files/PAR-20-035.html). Yet, not every potential interaction can be examined because of increasing research complexity, including potentially exponentially larger samples as more combinations are examined (compared to single substance studies). Furthermore, because it is recognized that studies of polysubstance use require complex designs and analysis, the grant program mentioned above includes special review (i.e., review by a specifically designated initial review group).

Priority areas where addressing polysubstance use is particularly urgent may include those combinations that are shown to have negative effects and population impacts. One specific combination for research is to understand the mechanisms of specific and apparently deadly combinations of opioids with alcohol, benzodiazepines, and other sedating agents, and some work to address these interactions is underway [95]. For other combinations, prioritization will be required. Fortunately, large-scale human studies such as the National Survey on Drug Use and Health, the Adolescent Brain Cognitive Development study, and other population studies are poised to provide data which can help to identify meaningful polysubstance use patterns [43, 96]. That is, these and other population studies can identify specific types of drug combinations that show particular relevance to human health for examination in basic research to determine mechanisms of action. Given that polysubstance use is more often the rule than the exception, novel approaches are needed to predict the multiple points of intersection of drugs that would result in a greater risk of a use disorder or toxicity from drug combinations. Likewise, novel approaches are needed to predict therapeutic measures that go beyond targeting a single drug–receptor interaction and toward the long-term plasticity that maintains addiction.

One promising approach relies on a big data approach of developing predictive models by computational mining of datasets related to drug abuse [97]. A centralized database of chemicals, target proteins, cellular signaling pathways, and tissues related to abused substances was recently generated from the literature and public databases, with the goal of identifying networks of interactions that would predict the consequences of polysubstance use, underlying mechanisms of these consequences, and potential means of targeting these mechanisms [98]. This approach has the potential to discover novel mechanisms of synergy by identifying common target proteins and convergent signaling pathways. As this is a relatively new approach, its utility will depend on the breadth of the database and sophistication of the computational tools, both of which will evolve with time.

We also highlight the potential importance of careful translation of preclinical research to human studies. For example, preclinical models of drug reward (i.e., Fig. 2) could be updated with clinical research to identify which pathways and receptors are related to different substances in humans. Some of this work has been started in the area of unique and general brain pathway correlates of addictions to various substances [99], but both new research as well as thoughtful graphical synthesis to illustrate the impacts are needed.

On a more immediate level, overdoses due to multiple substances may not respond fully to administration of naloxone, which targets only the opioid agents. Needed are both overdose-reversal agents for other substances and general approaches to support respiration. Such respiratory agents could be beneficial regardless of the overdose cause.

While sex and gender differences in polysubstance use have not been a major topic of research, sex and gender differences need to be considered in both the population and neurobiology studies. Patterns of opioid consumption and progression vary according to sex and gender and detailed analysis of these patterns for polysubstance use are needed as well to optimize individualized strategies for prevention and treatment. Additionally, sex differences can be used as a tool to elucidate the neurobiological mechanisms underlying polysubstance abuse.

## Conclusions

Interventions and research to address the U.S. opioid crisis have, for the most part, targeted opioid use, misuse, and addiction specifically. Even though it is the norm in most research on drug effects to look at substances in isolation, this greatly limits our understanding and risks ignoring important synergies, including potential additive (or multiplicative) effects of multiple substances on reward and reinforcement, as well as enhanced morbidity such as overdose. To account for polysubstance use, prevention approaches may benefit from targeting universal, common factors across substances [100], and treatment development needs to account for this polysubstance use as well. Treatment may need to focus on general factors, rather than drug-specific features. A target for treatment to address polysubstance use may be to focus on strengthening decision-making in general or targeting other common features across substances, such as craving or use of substances as a coping mechanism. Service delivery systems may need to be modified to address both general addiction issues that can inherently target multiple substances and to address the specific overlapping conditions that patients experience.

While polysubstance use patterns have been present for many years, as the number of drugs available to users increases (i.e., with development of more synthetic substances), there may be more current opportunities for polysubstance use than in the past, and more may be forthcoming, with resulting morbidity and mortality. The fact that many (and perhaps most) persons with an OUD use multiple substances (both over their life course and simultaneously in specific drug-using episodes) makes it imperative to learn more about polysubstance use and its consequences [39]. Consequently, research on the co-use of opioids with multiple other substances is needed across the spectrum of prevention and treatment interventions, overdose reversal, public health surveillance, and basic neurobiology.

## Disclaimer

The findings and conclusions of this study are those of the authors and do not necessarily reflect the views of the National Institute on Drug Abuse of the National Institutes of Health, the Centers for Disease Control and Prevention or the U.S. Department of Health and Human Services.
